# Research progress of 3D printing technology in the field of oral materials

**DOI:** 10.1016/j.pscia.2025.100093

**Published:** 2025-09-12

**Authors:** Khan Rajib Hossain, Dipika Ramdas Kalambhe, M. Abdul Jalil, Nusrat Tabassum Farah

**Affiliations:** a Materials Engineering, University of Chinese Academy of Sciences, Beijing, 100040, China; b Department of Natural Sciences, BGMEA University of Fashion and Technology, Dhaka, 1230, Bangladesh; c Shanghai Institute of Materia Medica, Chinese Academy of Sciences, Shanghai, 200120, China; d Public Health and Hospital Administration, National Institute of Preventive and Social Medicine, Dhaka, 1212, Bangladesh

**Keywords:** 3D printing, Oral materials, Prosthodontics, Orthodontics, Implantology

## Abstract

The emergence of three-dimensional (3D) printing has represented a significant technological breakthrough, exerting a profound influence across various domains of society in recent times. There has been a noticeable shift in the healthcare industry's dominant paradigm of therapeutic interventions, particularly regarding the utilization of 3D printing technology to mend or replace damaged or missing biological components. Adopting cutting-edge technology can significantly enhance the range of applications within the field of oral healthcare. A significant amount of research is being conducted to leverage the substantial potential of 3D printing in oral applications, with a focus on developing customized treatment plans tailored to specific case scenarios. By building specialized implantology from different biological composite materials layer by layer, 3D printing technology precisely restores the anatomical structure of defects in the mouth and maxillofacial region. This review presents a comprehensive discussion on the history and classification of 3D printing technology, as well as the dynamics of biological materials, cells, and bioactive factors utilized in repairing oral and maxillofacial bone defects. Additionally, this review provides an update on the materials commonly used in typical oral healthcare applications and examines future trends and concerns related to material perspectives in oral healthcare management.

## Highlights

• 3D printing enables customized oral implants, scaffolds, and prosthetics.
• Dentistry uses metals, ceramics, polymers, and bio-derived materials.
• Hydrogel and PRF bioprinting have potential for tissue regeneration.
• Cytotoxicity, mechanical weakness, and processing problems are some of the current drawbacks.
• Eco-friendly materials and customized digital dentistry are promising future developments.

## Graphical abstract

**Figure:**
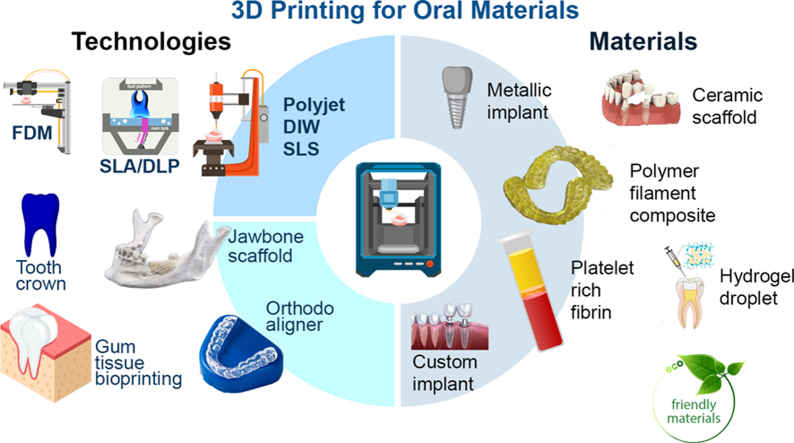

## Abbreviation list:

3D: Three-Dimensional
3DP: Three-dimensional Printing
3 ​MF: 3D Manufacturing Format
ABS: Acrylonitrile Butadiene Styrene
AM: Additive Manufacturing
BCP: Biphasic Calcium Phosphate
CAD: Computer-aided Design
CAM: Computer-Aided Manufacturing
CHA: Coral Hydroxyapatite
DIW: Direct Ink Writing
DLP: Digital Light Processing
ECM: Extracellular Matrix
FDM: Fused Deposition Modeling
LAB: Laser Assisted Bioprinting
MJ/Polyjet: Material Jetting
MSC: Mesenchymal Stem Cells
PBF: Powder Bed Fusion
PCL: Polycaprolactone
PFP: Powder Fusion Printing
PLA: Polylactic Acid
PLGA: Polylactic-co-glycolic Acid
PRF: Platelet Rich Fibrin
SCAP: Stem Cells from Apical Papilla
SEBM: Selective Electron Beam Melting
SFF: Solid Freeform Fabrication
SLA: Stereolithography
SLS/SLM: Selective Laser Sintering
STL: Standard Tessellation Language
STL: Standard Triangle Language
β-TCP: β-tricalcium Phosphate

## 1 Introduction

3D printing technology, also known as 3D rapid prototyping technology or additive manufacturing technology [1], is based on digital model data, utilizing various bondable materials, such as powdered metal, polymer, and biomaterials, to construct objects through layer-by-layer printing. Charles W. Hull first proposed this technology in 1986 [2]. Since then, with the increasing application of digital technology and the development of the manufacturing industry, 3D printing technology has made significant progress and has been successfully applied in various fields of oral medicine, including maxillofacial surgery, restoration, orthodontics, and tooth root canal preparation [3]. Compared to other fabrication methods, 3D printing technology offers several advantages, including greater accuracy, faster production, ease of use, and cost-effectiveness. Additionally, it provides versatility in printing multiple biological materials and creating structural objects with complex designs, either alone or in combination. The application of 3D printing technology in various disciplines is progressing rapidly. Among them, the application in the field of oral materials is mainly reflected in the following four aspects: Printing the tooth models according to digital image data to simulate clinical operations and teaching; Tooth or mandibular defects; 3D printing technology is applied to the field of tissue engineering and regeneration medicine, and stent materials with biological activity are used for regeneration research such as teeth, mandibular, and periodontal. The advancement of a diverse range of printed biomaterials has facilitated enhanced manipulation of both the internal structure and external form of scaffolds. The now accessible analytical digital instruments provide efficient and accurate means of capturing and recording the individualized patient condition in three dimensions. The seamless transmission of digital data facilitates the creation of anatomically precise structures that can be tailored to the unique specifications of individual patients [4]. The expiration of key patents related to 3D printing has led to a significant decrease in the price of printers. The swift advancements in these technologies have introduced novel and stimulating methodologies across various domains of medicine, including dentistry. Table 1 presents a chronological representation of the significant advancements in 3D printing technology, and Table 2 presents their respective medical uses.

**Table 1:** A timeline illustrates how three-dimensional (3D) printing technologies, crucial in the medical field, have evolved. [2]

Year	Key Developments
1984	The advent of stereolithography (SLA) 3D printing, credited to Charles Hull.
1999	The Wake Forest Institute for Regenerative Medicine successfully utilized a 3D-printed organ, specifically a bladder, for transplantation.
2005	The establishment of RepRap, an open-source endeavor aimed at constructing a 3D printer capable of autonomously producing a significant portion of its constituent parts.
2014	The initial instance of 3D-printed human liver tissue, developed by Organovo, and the advent of the first desktop bioprinter, pioneered by Allevi, are significant milestones in bioprinting.
2018	The Poieskin (Poietis) is the inaugural commercialization of a fully human tissue model, specifically in the form of 3D-printed skin.
2020	Customized medicine with a 3D printer, the M3DIMAKER (FabRx).

**Table 2:** Comparison of the main 3D printable oral biomaterials.

Material	Biocompatibility	Mechanical strength	Degradation rate	Applications
PLA [51]	Good, but degradation products may cause local acidic reactions.	Moderate	Medium to fast	Bone defect barrier membrane, implant scaffold.
PCL [50]	Excellent	High, good toughness.	Slow, up to >2 years.	Periodontal tissue engineering, long-term scaffolds.
PLGA [49]	Good	Adjustable, affected by molecular weight and ratio.	controllable, from weeks to months	Bone/periodontal regeneration, drug delivery systems.
Chitosan [31,47]	Excellent antibacterial properties.	Lower	Faster	Hemostasis, drug carrier, periodontal ligament repair.
Alginate [48]	Good binding to cells.	Lower	Medium	Cell/growth factor-loaded scaffolds.
Composites (e.g. PLGA/β-TCP, PCL/HA) [39,43]	Excellent, combining their respective advantages.	High, close to natural bone.	Aadjustable	Bone defect repair, implant scaffold

This review provides a summary of the application status of 3D printing technology in oral materials, including a discussion of the following four aspects: commonly utilized methods, basic stages, printable materials, and current issues.

## 2 3D printing technologies methods

**Fig. 1:**
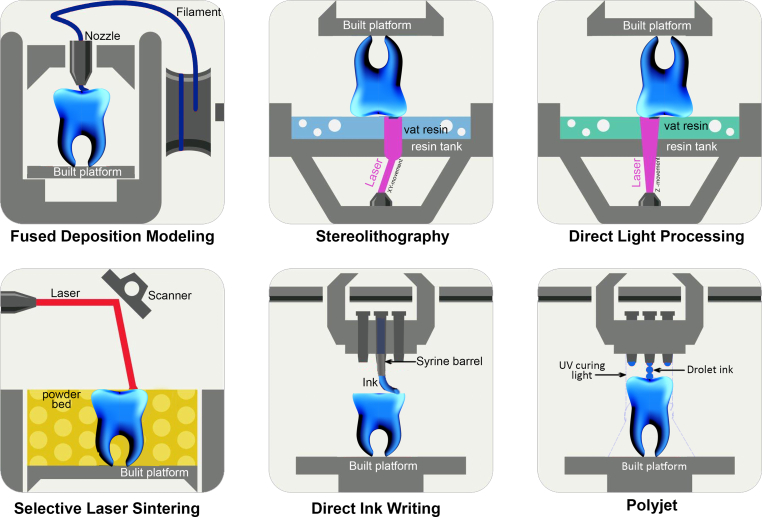
A schematic representation of the most often employed 3D printing methodologies (created using Adobe Illustrator): fused deposition modeling (FDM), stereolithography, digital light processing (DLP), selective laser sintering (SLS), direct ink writing (DIW), and material jetting (Polyjet).

The technique of 3D printing involves the automated deposition of materials in a sequential layer-by-layer fashion, producing a three-dimensional volumetric structure [5]. As with any nascent technology, implementing technical standards was essential to embrace a wide range of materials, commodities, systems, and services. The committee F42 on Additive Manufacturing (AM) technologies within the American Society for Testing and Materials (ASTM) and the International Organization for Standardization (ISO) has designated seven distinct categories for additive manufacturing. These categories are as follows: binder jetting, direct energy deposition, material extrusion, material jetting, powder bed fusion, sheet lamination, and vat photopolymerization [6]. In biomedicine, the prevailing technologies employed for printing can be classified into two primary categories: acellular methodologies and methodologies involving cellular components. Acellular approaches include stereolithography (SLA), powder-fusion printing (PFP), and solid freeform fabrication (SFF). Currently, the most widely used 3D printing methods in stomatology include fused deposition modeling (FDM) [7], selective electron beam melting (SEBM) [8], selective laser sintering (SLS) [9], stereolithography (SLA) [10], inkjet printing (poly jet printing) [11], and bioprinting (DIW) technology [12], etc (Fig. 1). The progressive vertical elevation of the receptacle, utilizing a polymer substance, leads to the gradual solidification of the material and the subsequent formation of a three-dimensional entity. The technique of selective laser additive manufacturing was employed for the fabrication of biodegradable polymers, ceramic acrylate, and hydroxyapatite materials, which are utilized in the field of bone restoration [13]. The application of lithography-based ceramics manufacturing (LCM) was employed in the fabrication of glass-ceramic dental restorations, with an emphasis on achieving a high degree of accuracy [14]. Zirconia implants were produced utilizing digital light processing (DLP), a 3D printing methodology that relies on photopolymerization [15]. Selective laser sintering (SLS) is a powder fusion printing (PFP) technology. In this process, small particles of metal, resin, or plastic are exposed to laser radiation, causing them to fuse in a step-by-step layering process [16]. In the present study, a tricalcium phosphate and hydroxyapatite approach was employed to fabricate scaffolds intended for bone regeneration [17]. One notable benefit of utilizing Powder Bed Fusion (PBF) techniques is its capability to facilitate printing various melting metals, including titanium, magnesium, and cobalt chromium, which have extensive use in medicine and dentistry. Solid form fabrication (SFF) involves the controlled deposition of strands through a nozzle facilitated by a highly accurate positioning mechanism along the XYZ axes. However, the material must maintain its shape upon extrusion. For instance, the researchers mixed polycaprolactone (PCL) with alginate to fabricate scaffolds intended for cartilage repair [18].

The technique of incorporating cells into the process of 3D printing has been officially named “bioprinting”. In contrast, the hydrogels used as the medium for cell deposition during the printing procedure are referred to as “bioinks” [19]. Hydrogels exhibit the capacity for a modifiable chemical composition, along with the ability to alter their mechanical and biodegradation properties [20]. Hydrogels are often regarded as highly advantageous materials for bioinks due to their biocompatible properties, minimal cytotoxic effects, and substantial water content [21]. For a hydrogel to be considered suitable for 3D bioprinting, it must exhibit sufficient viscosity to maintain its structural integrity during the printing process without causing adverse compaction of the enclosed cells. Furthermore, the hydrogel must possess cross-linking properties since this would facilitate the retention of its three-dimensional architecture after printing. Extrusion bioprinting encompasses the process of extruding filaments by using either pneumatic (pressure-driven) or mechanical (plunger-driven) forces. Rapid gelation serves to preserve the desired outward form and interior arrangement. As an illustrative example, the combination of alginate with calcium and fibrinogen with thrombin might be cited. One significant advantage of extrusion bioprinting is its ability to utilize various materials and cell types in varied combinations [22]. Laser-assisted bioprinting (LAB) operates by using a laser pulse to induce localized heating of a solution containing cells, resulting in the precise placement of cells on a predetermined region of a platform or substrate [23]. The laser-direct-writing approach, a subset of laser-assisted bioprinting (LAB), has demonstrated successful utilization in depositing a wide range of cell types and biomaterials [24]. In inkjet bioprinting, a predetermined volume of fluid, whether containing biological constituents or not, is ejected onto a substrate to achieve high precision in the arrangement. Droplets are deposited by harnessing either thermal or piezoelectric energy. One noteworthy advantage is the speed at which complex tissue constructs may be fabricated, containing cells and mimicking natural tissues. This is facilitated by a multi-head bioprinting approach, which enables the simultaneous deposition of many cell types and biomaterials. One significant constraint involves the need for cells or bioactive substances to be in a liquid state, allowing for their deposition and subsequent solidification into the intended configuration. Hydrogels commonly utilized in laboratory methodologies are cross-linked by various mechanisms, including physical, chemical, pH, and UV light methods [25]. A comprehensive analysis of the two distinct categories of 3D printing techniques relevant to tissue constructs has been presented in a recent scholarly work [26].

FDM is the earliest 3D printing method used in clinical medicine and stomatology. It has the advantages of a wide application range, reliable printing quality, convenient installation, and low cost. It can print materials such as acrylonitrile butadiene styrene (ABS) and polylactic acid (PLA). The basic process of FDM involves feeding printing material into a hot nozzle after melting, extruding it into a two-dimensional plane, and printing layer by layer as the nozzle is raised or the printing bed is lowered. In comparison, FDM is more suitable for printing models with relatively simple anatomical structures, such as models of teeth or various restorations, teaching aids, etc.; for objects with very complex design structures, it has the disadvantages of longer printing time, limited color selection, lower printing accuracy, and demanding removal of support materials [27]. SEBM is a powder bed fusion 3D printing technology that was developed in the mid-to-late 1990s. This technology operates in a vacuum environment, offering significant advantages, including a high energy utilization rate, a fast scanning speed, and low forming stress. It is particularly suitable for directly forming active metal materials that have high melting points or are brittle and difficult to process [28]. The basic process is as follows: (1) Slicing and layering the designed object according to a specific thickness to obtain all two-dimensional information; (2) Using electron beams as the energy source in a vacuum environment and controlled by a computer under the action of an electromagnetic deflection coil, scanning and melting the powder layer laid on the workbench according to the CAD data of each layer section parts. Titanium alloy implants prepared by SEBM technology have been successfully utilized in medical orthopedic implant materials, and porous tantalum and cobalt-chromium alloys have also undergone clinical testing. The future market is expected to exhibit an explosive growth trend [29].

At present, the application of the inkjet printing method in the medical field includes processing personalized 3D models of human tissues and organs with complex anatomical structures and then determining the surgical plan; processing brackets or guide plates required during surgical operations; preoperative simulation of plastic or cardiac surgery; 3D printing for tissue engineering scaffolds, etc. Over the past decade, the rapid development of 3D printing in various disciplines has led to the integration of 3D printing technology with tissue engineering and regenerative medicine, resulting in the emergence of bioprinting technology [30]. Scaffolds that can be loaded with cells have been created using a variety of materials, such as calcium silicate composites, chitosan, and polymer materials that release bioactive factors gradually. In cell culture, bioprinting technology enables the creation of a three-dimensional cell culture system for tissue engineering, drug screening, and the development of in vitro disease models by combining seed cells with 3D-printed polymer scaffold materials. Currently, bioprinting technologies utilize “cell ink” or cell aggregate/microtissue systems to fabricate artificial “tissues”. These technologies have demonstrated the ability to generate intricate 3D structures in laboratory settings. The introduction of 3D printing has also accelerated the development of clinical stem cell therapy technology. With the help of this novel technique, seed cells can be precisely deposited onto specially designed scaffold materials, making it easier to transplant them into living organisms for tissue regeneration and repair [31].

### 2.1 Basic steps of the 3D printing process

**Fig. 2:**
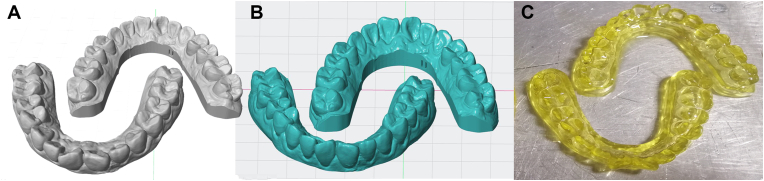
(A) The first step in the process is designing the product with SolidWorks 3D CAD software, which produces a digital model of the required 3D object. 3D model is exported as a 3 ​MF (3D Manufacturing Format) or STL (Standard Tessellation/Standard Triangle Language) file, which includes the 3D printer's geometric information, (B) Importing the STL or 3 ​MF file, the model is divided into thin, horizontal layers using UnizDesktop slicing software, which also creates G-code instructions for the 3D printer, and (C) Chosen material is deposited and fused layer by layer by the UnizDesktop 3D printer following the G-code instructions, creating the 3D object.

The three steps of the entire 3D printing process are as follows: using scanned or computed tomography data, create a digital 3D model in the software; analyze and process the 3D model to form many “two-dimensional layer” data after “layered cutting”; build a solid model of the object by layering up materials; print the final 3D object layer by layer; and process the printed object after printing, including processing the printed object and carefully removing the support structure (Fig. 2). This fundamental process is compatible with a variety of 3D printing technologies and materials, including metals, ceramics, and polymers [32].

## 3 3D-printed oral materials

Currently, 3D printing technology has permeated all aspects of social life, and numerous extensive studies have been conducted on 3D printing materials. This article mainly introduces the 3D printing materials involved in oral materials. Such materials are required to have apparent cytotoxicity and good biocompatibility. For 3D printing materials used in dental pulp, periodontal regeneration, and other applications, it is necessary to promote biological behaviors such as cell adhesion, proliferation, and differentiation on the material's surface. The five main categories it includes are metals, bioceramics, biodegradable natural and synthetic polymers, and materials derived from humans, including blood and teeth [33].

### 3.1 Metal material

Metallic biomaterials are widely used in oral and plastic surgery because of their excellent mechanical properties. Compared to polymers and ceramics, it exhibits high strength, toughness, and hardness, making it more suitable for load-bearing applications. According to reports, the mechanical properties of metal stents can be changed by adjusting the pore size of metal materials. High biocompatibility, suitable mechanical properties, and elasticity are key characteristics of titanium and titanium alloys that support bone regeneration. High mineral deposition is linked to the good hydrophilicity of titanium-based 3D scaffolds. This was confirmed by in vitro experiments, which demonstrated that the scaffolds promote cell adhesion, proliferation, and new bone formation without any signs of inflammation or necrosis in vivo. Intraosseous implants cannot be used as carriers for bioactive molecules or as a substitute for titanium implants, despite titanium's proven suitability as a material. The lack of biodegradability of titanium and titanium alloys, however, is a significant drawback that necessitates a second surgery for removal, thereby significantly increasing medical expenses [34].

**Fig. 3:**
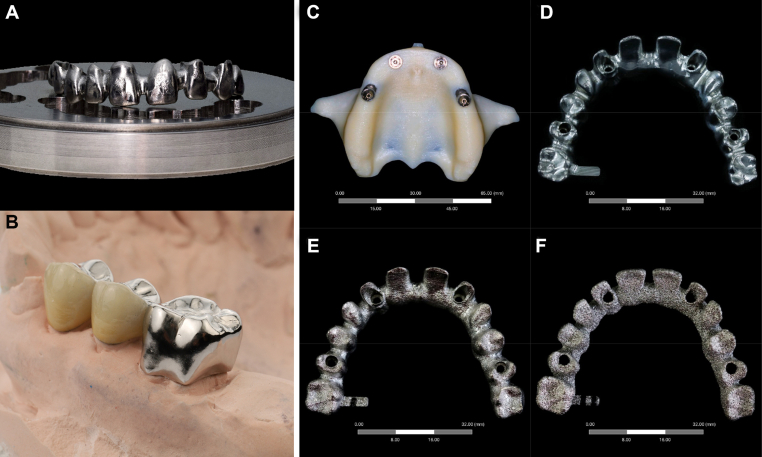
3D printed dental teeth (A) biocompatibility of porcelain fused to titanium, (B) Gold alloys implant crowns, (C) developed a prototype master model incorporating implants and corresponding abutments, (D) milling framework, (E) additive selective laser melting (SLM) framework, and (F) additive electron beam melting (EBM) framework. Copyright permission from Ref. [40], [2020],[Elsevier].

Researchers have studied magnesium and its alloys in great detail over the past decade, discovering that magnesium ions can promote osteogenic differentiation and cell adhesion. Magnesium alloys are a hotspot for medical material research because their elastic modulus is comparable to that of human bones, and they combine the benefits of both inert and biodegradable materials. Although pure magnesium degrades rapidly in the body, it can be controlled using magnesium alloys or coating pure magnesium with titanium or ceramics [35]. Gao et al. [36] conducted a comparative analysis of the alloy's structure and properties before and after hot isostatic pressing. They found that the thermal densification of the 3D printed magnesium alloy using the hot isostatic pressing process can significantly refine the alloy structure to a certain extent. The mechanical properties and wear resistance have been improved considerably. Ye et al. [37] prepared magnesium-based bone nails by hot isostatic pressing after 3D printing and conducted microstructure observation and experimental analysis of mechanical properties and biocompatibility. The magnesium-based bone nails were found to be dense, small, and have no noticeable pores. They have similar mechanical properties to those of human bones and exhibit better biocompatibility. Compared to titanium alloys, cobalt-based alloys offer high hardness, excellent wear resistance, and a lower cost. They are more suitable for manufacturing long-term implants that undergo harsh loading conditions within the body. They are also commonly used metals in medical treatment. However, their application is limited due to the severe sensitization of cobalt and nickel elements. However, in recent years, surface modification technology has improved its clinical application effect. Tantalum, niobium, and zirconium have good chemical stability, physiological corrosion resistance, and biocompatibility. They have also been used in biomedicine, but their high prices limit their wide application. This group of materials can be used in the repair of maxillofacial bone defects, tooth restoration, implant manufacture, and stomatology. Each material has notable characteristics and limitations. However, since hard tissues, such as bones and teeth, are composed of both organic and inorganic components, it is challenging for a single biomaterial to replicate their complex tissue structure, and it is also difficult to meet all the characteristics required by the tissue. Therefore, in the application, two or more different biomaterials are often combined to produce a “synergistic effect” on the overall performance, thereby improving the scaffold's mechanical properties, biological activity, and degradation ability. These materials are referred to as “composites” or “hybrids,” such as “ceramic/metal” and “polymer/metal.” [38]. Zhu et al. [39] introduced a multi-parameter research study on polylactic-co-glycolic acid (PLGA)/β-tricalcium phosphate (β-TCP)/magnesium (Mg) porous scaffolds. Using polymer/ceramic/metal composite materials, the problems with degradable materials currently used for repairing bone defects include a lack of influential interactive factors, unsuitable mechanical strength, and a high risk of local inflammation. The osteogenic activity of the porous scaffold is improved to facilitate bone regeneration and functional reconstruction at the implanted site (Fig. 3).

### 3.2 Bioceramic materials

Bioceramics, such as bioactive glass and calcium phosphate bioceramics, are frequently utilized as bone fillers in orthopaedics and oral cavities [41]. Biphasic calcium phosphate (BCP), tricalcium phosphate (TCP), and hydroxyapatite (HA) are examples of calcium phosphate bioceramics. The most extensively researched calcium phosphate bioceramic, HA, is osteoinductive because it shares the same chemical composition as natural bone and teeth, promoting osteoblast adhesion and proliferation. HA is non-absorbable, which is beneficial for maintaining the stability of the bone regeneration space [42]. There are two types of TCP: a high-temperature type α phase and a low-temperature type β phase. Yeo et al. [43] demonstrated that β-TCP promoted new bone formation when degraded in vivo, and its biodegradability was the strongest among TCPs without hindering the formation of the bone matrix. The physicochemical and biological properties of β-TCP are superior to those of HA. After being implanted into the body, it can degrade itself and maintain the solid-liquid balance of calcium and phosphorus ions near the bone graft bed. These calcium and phosphorus ions eventually form calcium and phosphorus solids and deposit on the bone surface, forming a direct bond with the bone.

**Fig. 4:**
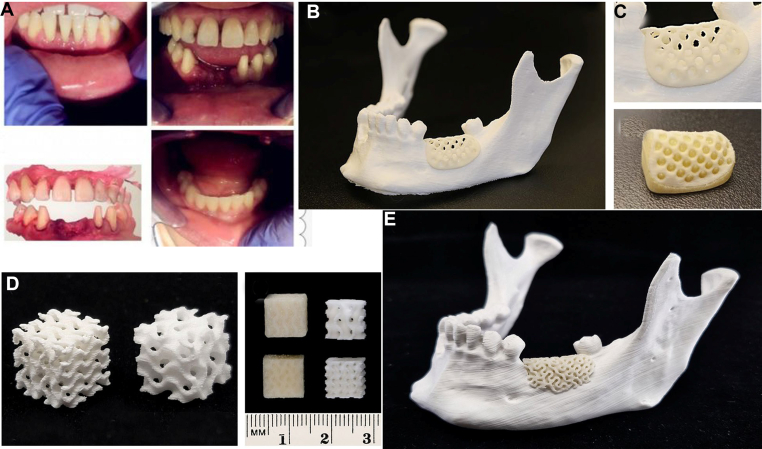
(A) 3d printed multilayer zirconia-based teeth, (B) 3D printed mesh (thickness 1.0 ​mm) for the guided regeneration of alveolar bone. Visual evaluation of the implant's compatibility with the 3D-printed replica. (C) enlarged perspective of the open-porous alveolar network, and integrate with the support structure before post-processing, (D) bioresorbable composite scaffolds are depicted in isometric and frontal views, illustrating the scaffolds before (left) and after (right) the removal of the water-soluble support material, and (E) Patient-specific implant (PSI) bone graft for alveolar bone deficiency, affixed to a 3D-printed mandible. Copyright permission from Ref. [46], [2024], [Woodhead Publishing].

BCP is produced when the two-phase component of TCP is combined with HA. Compared with other calcium phosphate ceramics, BCP has more significant biological activity, mechanical properties, and the ability to promote bone growth. BCP is a suitable material for use as a bone graft substitute because it exhibits osteoconduction and good tissue compatibility. BCP is widely used in periodontics, orthopaedics, and oral and maxillofacial surgery due to its low cost and high output. BCP is a safe, biocompatible scaffold that supports new bone formation, alone or combined with growth factors. Tsai et al. [44] employed the gel method to develop a magnesium-calcium silicate cement (Mg-CS) scaffold material that enables rapid solidification and controlled degradation, thereby regenerating hard tissues such as bones and teeth. The study found that the degradation rate of Mg-CS depends on its Mg content. The slow release of Mg ions can promote the proliferation ability of periodontal ligament cells, increase the secretion of alkaline phosphatase (ALP), and enhance the expression of tooth generation-related genes DSPP and DMP-1, as well as angiogenesis-related proteins vWF and ang-1, confirming that the material has the functions of promoting tooth generation and angiogenesis. The above materials can also be used as injectable cement (paste). This plastic calcium phosphate material exhibits good adaptability to bone defects with complex shapes, which is a challenge to achieve with traditional bone graft materials. Bioceramic materials are widely used in bone and dental tissue reconstruction due to their excellent biocompatibility, bioactivity, hydrophilicity, osteoconductivity, potential osteoinductive ability, and composition similar to that of natural bone, as well as their relatively low cost. However, due to its disadvantages of poor rigidity, low toughness, and fragility, it isn't easy to shape and design objects with complex structures. SLS technology in 3D printing can compensate for some of its defects that are difficult to shape, allowing it to be applied in bone and oral tissue repair; however, it is still limited to non-load-bearing areas [45] (Fig. 4).

### 3.3 Biodegradable natural polymers

Natural polymers, including proteins and polysaccharides, are the first biomaterials utilized in various clinical applications due to their excellent hydrophilicity, biocompatibility, cell recognition, and ability to enhance cell interactions within the surrounding environment. These characteristics have led to their extensive exploration as hydrogels in the study of scaffold materials for tissue engineering, with positive outcomes. One of the most abundant proteins in the body, collagen gives different tissues, such as skin and bone, their strength and structural stability. Polysaccharides and chitosan exhibit good hemostatic qualities, non-toxicity, biodegradability, pain alleviation, antibacterial activity, wound healing, and biocompatibility. These properties can significantly reduce the risk of stent contamination and postoperative infection, thereby preventing the final exposure of the stent and surgical failure [47]. Alginate and chitosan do not exist in the human body. Glycosaminoglycans (GAGs), which are excellent materials for tissue regeneration, are structurally similar to those found in the natural extracellular matrix (ECM) of human tissues, including bone, according to the authors. Its disadvantages are the lack of biological activity, weak mechanical properties, and fast degradation rate under enzymatic reactions, which limit the application of natural polymers in the regeneration of oral tissues [48].

### 3.4 Biodegradable synthetic polymers

**Fig. 5:**
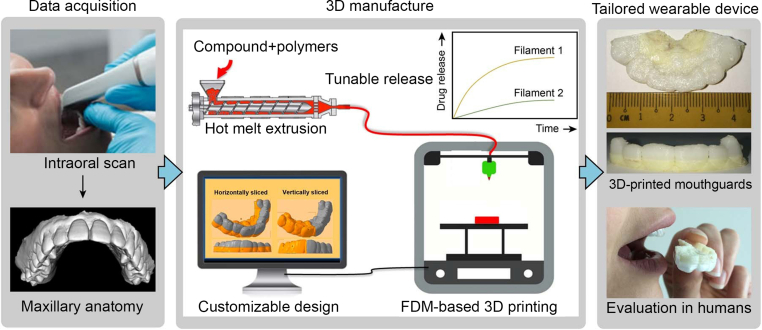
Creation of personalized mouthguards for oral delivery using 3D printing technology. There were two stages involved in creating personalized oral delivery mouthguards using 3D printing. The subject's maxillary structure was scanned intraorally during the data collection process, and the impression was used as the 3D printing template. The development of printable pharmaceutical-grade filaments infused with the necessary chemical using hot-melt extrusion marked the beginning of additive manufacturing. Obtained filaments with variable release rates by altering the feed's polymer composition, and then used them to produce prototypes using FDM-based 3D printing that had customizable designs (derived from scanned templates). Copyright permission from Ref. [52],[2018],[AAAS].

Due to the relatively low cost of biodegradable synthetic polymers, they have a longer shelf life than natural polymers. The most studied biomaterials in this group are aliphatic polyesters, including polycaprolactone (PCL), polylactic acid (PLA), polyglycolic acid (PGA), and poly(lactic-co-glycolic acid) (PLGA), among others. Among them, PCL exhibits characteristics of good biocompatibility, a slow degradation rate, and high mechanical stability, and making it suitable for various scaffold manufacturing technologies [49]. Suárez-González et al. [50] designed PCL scaffolds with different pore sizes, loaded with periodontal ligament stem cells, and used them for the regeneration of the periodontal complex (bone/periodontal ligament/cementum) and found that the pores with different pore sizes designed by 3D printing have the characteristics of inducing various types of tissue regeneration. In general, aliphatic polyesters degrade more slowly than natural polymers and bioceramics. Synthetic polymers degrade by hydrolysis and are divided into bulk degradation and surface erosion. Most available polyesters are hydrolyzed by bulk degradation, a mechanism characterized by hydrolysis within the biomaterial, leading to empty shells but retaining the outer dimensions for a considerable period. This feature makes them suitable for scaffolds for bone graft substitutes, but less suitable as carriers for drug delivery. Sandberg et al. [51] applied PLA membranes to the study of bone defects and found that osteoblasts in the periosteum proliferate, differentiate, and secrete bone matrix to calcify bone along the surface of the PLA membrane; at the same time, PLA materials can stimulate periosteal osteogenesis through their degradation products (Fig. 5).

### 3.5 Human teeth, blood, and other derivatives

**Fig. 6:**
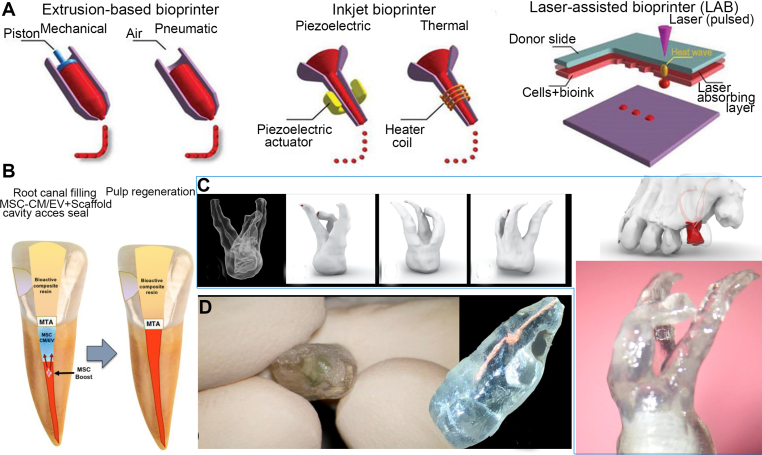
(A) Principal methodologies in bioprinting, (B) A cell-free methodology for dental pulp regeneration utilizing mesenchymal stem cells (MSC) produced conditioned media/exosomes. The essential components involved are MTA (mineral trioxide aggregate), SCAP (stem cells from apical papilla), and EDTA (ethylenediaminetetraacetic acid), Copyright permission from Ref. [55],[2023],[MDPI] (C) data from cone beam computed tomography (CBCT) were uploaded to stereolithographic (STL) software, and a transparent plastic model was produced using 3D printing, and (D) 3D models of teeth were fabricated, and primary root canal therapy and retrograde filling via invagination were performed. Copyright permission from Ref. [56],[2021],[MDPI].

Extracellular matrix (ECM)-derived scaffolds can provide a natural and cytocompatible microenvironment for tissue regeneration. Among them, the dentin matrix has been shown to contain a variety of soluble and insoluble signalling molecules that promote tooth formation. Athirasala et al. [53] developed a new type of bio-ink, which mixed the printable alginate hydrogel with the soluble and insoluble parts of the dentin matrix. They discovered that the soluble dentin molecules were better preserved and that the hydrogel mixture's viscosity was more suitable when the alginate to dentin ratio was 1:1. According to the authors, this bio-ink has a natural odontogenic ability and good cytocompatibility, which makes it appropriate for both tooth regeneration and the creation of tooth-morphological scaffolds with intricate three-dimensional microstructures. Platelet-rich fibrin (PRF) is a second-generation blood concentrate product that can effectively stimulate the regeneration of both soft and hard tissues. It contains a variety of platelets and cytokines. It has the advantages of being low-cost and easy to prepare. Li et al. [54] compared the osteogenesis effect of the coral hydroxyapatite (CHA) and PRF compound with that of CHA alone and found that the former was significantly better. A photosensitive resin scaffold was created using 3D printing in a clinical treatment overseas using the CBCT data set of a tooth with a periodontal defect before surgery. The patient's blood was centrifuged to extract PRF, combined with Bio-Oss bone powder, and then loaded into the scaffold. The upper portion of the scaffold was then covered with PRF membrane and Bio-Gide collagen membrane. The 15-month follow-up revealed that the depth of the periodontal pocket at the treatment site had decreased, and a small amount of new periodontal bone tissue had also formed. PRF is regarded as an ideal biomaterial for periodontal regenerative treatment (Fig. 6).

## 4 Problems faced by 3D printing technology in the application of oral materials

Subtractive manufacturing (e.g., CNC milling), high machining accuracy, and good surface quality are suitable for metal and ceramic restorations, but limited design freedom and high material waste. Casting processes are ideal for large-scale production of metal restorations, but have long cycle times and low personalization. Injection moulding/compression moulding is low-cost, suitable for large-scale production of polymers and ceramics, but challenging to achieve complex anatomical structures. We have emphasized the practical clinical applications of these processes and compared them with 3D printing, highlighting their unique advantages in personalization, complex structure manufacturing, and rapid prototyping. Although commercial 3D printing, such as inkjet printing, has achieved extremely high resolution in recent years, the properties of printable materials still need to be severely restricted to enable the application of 3D printing technology to implantable oral biomaterials. Organic solvents will not only damage the biocompatibility of the scaffold but also damage the service life of the 3D printer head [57,58]. Therefore, developing water-based adhesives for stent fabrication is recommended to minimize the use of organic solvents. Higher processing temperatures will damage and shorten the working life of ordinary 3D printers. Therefore, the feasibility of low-temperature 3D printing should be explored, especially for ceramic materials, which can enhance their stability, control shrinkage, and add bioactive factors, polymers, etc [59]. Currently, the most widely used 3D printing technologies cannot maintain the survival of cells and the activity of biomolecules for an extended period. Suppose the limitations of the above technologies are overcome. In that case, adding living cells, growth factors, or drugs to the 3D-printed scaffolds can further enhance the application of 3D printing technology in oral regeneration [60]. This ensures that the printed scaffold material has good mechanical properties, high resolution, and a precise porous interconnection structure simultaneously. This problem can be solved by preparing a mixture of composite biomaterials and optimizing the treatment process after 3D printing. By coordinating the mechanical properties, biocompatibility, drug delivery ability, and degradation characteristics of printed scaffold materials, the various properties of 3D printing materials can be improved [61,62].

## 5 Clinical translation

The clinical applications of 3D printing technologies in dentistry have made significant progress in recent years, and numerous interesting examples show a concrete application of it in OMFS, Prosthodontics, and Orthodontics. Patient-specific implants and surgical guides produced with DLP or SLM techniques have already reached clinical trials in maxillofacial reconstruction and implantology and led to higher accuracy, less intraoperative time, and better patient outcomes. For instance, additively manufactured patient-specific titanium or zirconia implants are being evaluated for clinical osseointegration and long-term stability, and promising results are being seen [63]. Moreover, 3D-printed hydroxyapatite, β-TCP, and biodegradable polymer scaffolds have also been clinically investigated, as well as in case studies of alveolar bone defect treatment, which indicates good biocompatibility and bone regeneration [64].

3D printing in prosthodontics is becoming more and more used for the making of crowns, bridges, and dentures due to its precision and speed in acquiring the final product. The integration of intraoral scanning, CAD/CAM-supported design of prosthesis, and additive manufacturing produced greater precision, both in the shaping of the prosthesis and in the aesthetic result, adapting to the patient-specific details. Customized clear aligners/orthodontic appliances made by stereolithography are currently being used as an efficient and practical move from translational system integration. Although successful in these applications, the scaling of bioprinting for soft oral tissues like gingiva and pulp regeneration is still facing challenges, where cell-laden hydrogels and bioactive scaffolds are still mainly on the preclinical and early trial stages. Overcoming regulatory obstacles, long-term efficacy, and cost efficiency are factors that will drive the generalization of these two approaches to routine dentistry [65].

## 6 Future prospectus

3D printing technology holds enormous promise in dentistry, oral health, and the development of oral materials. Some significant potential opportunities are as follows: There will likely be an increase in the use of customized dental implants and prosthetics, including crowns, bridges, and dentures. By utilizing 3D printing technology, dental solutions can be produced with precise dimensions, forms, and colors, thereby providing patients with greater physical comfort and aesthetic value [66]. The advancement of 3D printing technology may enhance oral surgery techniques for bone repair and regeneration. The application of 3D-printed scaffolds made from biocompatible materials is a promising technique for restoring bone tissue in areas damaged by trauma or decay [67]. Three-dimensional (3D) printing technology has the potential to completely change the manufacturing of transparent aligners, retainers, and other orthodontic products. The technology enables shorter production schedules and more accurate fittings, which enhance patient comfort and treatment effectiveness [68].

**Fig. 7:**
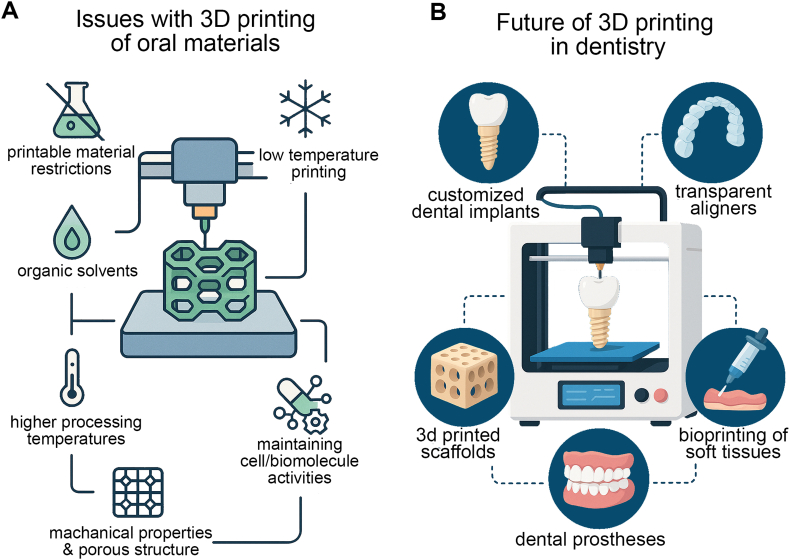
Challenges and potential applications of 3D printing in dentistry (prepared using SolidWorks and Adobe Illustrator). (A) The compatibility of biomolecules, temperature limitations, and material limitations are the primary challenges associated with 3D printing oral materials. (B) Custom implants, transparent aligners, 3D printed scaffolds, dental prostheses, and bioprinted soft tissues are examples of emerging applications in the field of regenerative medicine.

The process of bioprinting soft tissues and advancements in 3d bioprinting technology may make it possible to print biological tissues, including soft oral tissues like gums. The findings above may have a significant impact on the management of periodontal diseases and other oral health issues. Dental prostheses and devices can be manufactured much more quickly and cheaply thanks to 3D printing technology. This has the potential to improve the efficiency and affordability of dental care, benefiting both patients and dental offices. 3D printing technology in digital dentistry has the potential to expedite dental procedures, from the diagnostic stage to the treatment phase, when combined with other state-of-the-art technologies, such as intraoral scanners and computer-aided design/computer-aided manufacturing (CAD/CAM) systems. Future studies can focus on environmentally friendly strategies, such as using biodegradable materials for specific dental applications. Although significant advances have been made, there are still several limitations in the current studies of 3D printing in oral materials. In addition, no printable biomaterials can accommodate the high requirements on mechanical strength, biocompatibility, controlled degradation, and long-term cell viability and bioactivity simultaneously. Moreover, most of the studies are still pre-clinical, such as in vitro or small animal experiments, and there is a lack of large clinical trials and long-term follow-up studies. Furthermore, the lack of standardisation of evaluation methods and regulatory barriers prevents the comparison of outcomes across studies, which has hindered clinical translation. Consequently, future research will contribute to the fabrication of advanced composite biomaterials with adjustable properties, improvement of the process of bioprinting to stimulate cell survival and functional incorporation, and strict clinical investigation. The regulation and evaluation of 3D-printing technology for oral medicine will need to establish a set of consistent evaluation criteria between different organisations in order to make it possible to widely promote the safe and effective use of 3D printing in oral healthcare [69] (Fig. 7).

## 7 Conclusion

The utilization of 3D printing technology exhibits exceptional precision and effectiveness in producing a diverse range of materials. There has been a significant amount of research and practical application in the areas of oral and maxillofacial surgery, periodontal disease treatment, oral rehabilitation, and dental implantology therapy. Several time-sensitive issues require rapid resolution. Additional research is needed in the field of regenerative medicine, particularly in the area of identifying and utilizing suitable progenitor cells and biomaterials to promote regeneration of the jaw or periodontium. The investigation of techniques that maintain the physiological functions and functionality of different seed cells after they have been integrated into biomaterials is an important area that needs more research. Additionally, research techniques that aim to enhance the interaction between seed cells, bioscaffold materials, and the surrounding transplantation environment are fundamental. It is essential to be aware that there is a limited body of domestic scholarly investigations relevant to the application of 3D printing technology in oral materials and that the number of clinical trials conducted is also insufficient. It is anticipated that a larger number of domestic scientific research teams will participate actively in various comprehensive and rigorous research efforts. The application of 3D printing technology will soon bring about a significant transformation in the field of oral materials science.

## CRediT authorship contribution statement

**Khan Rajib Hossain:** Writing – original draft, Software, Resources, Conceptualization. **Dipika Ramdas Kalambhe:** Writing – review & editing. **M. Abdul Jalil:** Writing – review & editing. **Nusrat Tabassum Farah:** Writing – review & editing.

## Ethics approval

Not applicable.

## Declaration of generative AI in scientific writing

No generative AI tools have been used throughout the entire writing process of this manuscript.

## Funding information

There was no agency funding used in the drafting of this manuscript.

## Declaration of competing interest

The content of the article is free of pertinent conflicts of interest and is not sponsored by any manufacturer or associated business, either directly or indirectly. There aren't any confidentiality leaks, disagreements over other people's projects or patented technologies, duplicate submissions of the same manuscript, or authorship disputes in the content.

## Data Availability

Not applicable.
